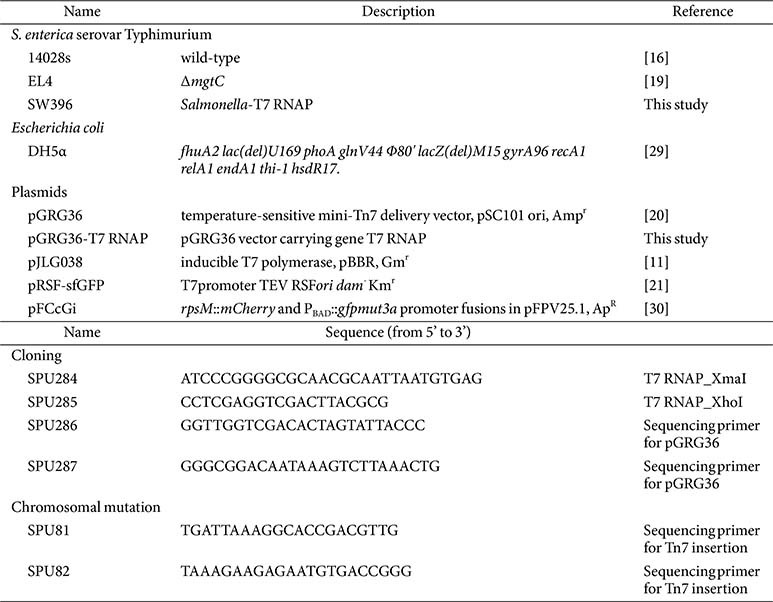# A Chromosomally Integrated T7 RNA Polymerase Enables T7-Derived Expression in *Salmonella enterica* without Compromising Virulence

**DOI:** 10.4014/jmb.2509.09023

**Published:** 2025-11-18

**Authors:** Seungwoo Baek, Seoyeon Kim, Eun-Jin Lee

**Affiliations:** Department of Life Sciences, School of Life Sciences and Biotechnology, Korea University, Seoul 02841, Republic of Korea

**Keywords:** *Salmonella*, T7 RNA polymerase, heterologous protein expression, pET vector system, chromosomal integration, host-pathogen interaction

## Abstract

The T7 RNA polymerase (T7 RNAP) system has revolutionized protein expression in *Escherichia coli* due to its high transcriptional activity and tight regulation. However, *Salmonella enterica*, despite its close genetic similarity to *E. coli*, lacks a T7 RNAP system, limiting the use of T7-based vectors and tools in this pathogen. Establishing a T7-compatible *Salmonella* strain would enable the seamless application of *E. coli*-optimized expression systems for studies in a pathogenic context. We engineered *S. enterica* serovar Typhimurium strain 14028s to stably express T7 RNAP from the chromosome under the control of the lac promoter using the pGRG36 transposon system. The resulting strain (*Salmonella*-T7) supports robust IPTG-inducible expression of heterologous proteins from T7 promoter-driven vectors, such as the pET series. *Salmonella*-T7 exhibited growth kinetics comparable to wild-type *Salmonella* in both rich and minimal media, indicating no detectable fitness cost. Furthermore, macrophage infection assays and murine infection models demonstrated that chromosomal integration of T7 RNAP does not compromise virulence. The engineered *Salmonella*-T7 strain enables efficient use of T7-based expression systems in *S. enterica* without affecting bacterial physiology or pathogenicity. This platform provides a valuable tool for studying bacterial pathogenesis as well as applications in synthetic biology and vaccine development.

## Introduction

T7 RNA polymerase/promoter system has revolutionized protein production in *Escherichia coli* due to its exceptional transcriptional activity and tight regulation [1-3]. Therefore, vectors such as the pET series, which exploit T7 promoters, are extensively applied in molecular biology and biotechnology for efficient expression of recombinant proteins [4-8]. Yet, purification of heterologous proteins expressed in *E. coli* inherently results in their dissociation from their native physiological conditions [9], thereby losing endogenous modifications and regulatory influences. For example, even though *Salmonella* is closely related to *E. coli* in terms of phylogeny, expression of orthologous *Salmonella* genes in *E. coli* is often inefficient due to differences in regulatory and folding contexts, leading to poor protein yield and functionality [10].

To investigate proteins within their native context, researchers often rely on ectopic expression in the native host, typically by inserting regulated promoters such as the lac promoter (p*_lac_*) in the chromosome. A two-part broad-host-range (BHR) plasmid system for T7 expression has also been developed [11]. While such systems can drive expression, they have limitations. Protein yields in the native host are often low because chromosomal expression systems fail to achieve sufficient expression levels. In single-vector expression systems, leaky expression often prevents tight regulation of the target protein. Although bipartite systems employing two plasmids can achieve tighter control, they require dual antibiotic selection, which is not ideal for studies involving pathogenic bacteria and their virulence. In *Salmonella enterica*, a widely used model organism for studying host-pathogen interactions [12, 13], it has been shown previously that heterologous expression under the control of the lac promoter compromises *Salmonella* virulence [14, 15].

In this study, we aimed to address these limitations by integrating T7 RNA polymerase into the chromosome of *S. enterica*. This engineered strain, hereafter referred to as *Salmonella*-T7, was designed to allow seamless use of existing T7-based vectors, such as the pET series, in a pathogenic *Salmonella* background. We demonstrate that *Salmonella*-T7 supports robust IPTG-inducible expression of heterologous proteins from pET vectors. Furthermore, because maintaining pathogenicity is essential for studying host-pathogen interactions, we evaluated whether this modification affects bacterial virulence. Our results show that *Salmonella*-T7 retains full virulence in macrophage infection assays and mouse models, establishing it as a powerful platform for both *in vitro* and *in vivo* studies of *Salmonella* physiology and heterologous protein expression in a pathogenic context.

## Materials and Methods

### Bacterial Strains, Oligonucleotides, and Growth Conditions

Bacterial strains, plasmids and oligonucleotides used in this study are listed in Table 1. All *Salmonella enterica* serovar Typhimurium strains are derived from the wild-type strain 14028s [16]. Bacteria were grown at 37°C in Luria-Bertani broth (LB), or in N-minimal medium (pH 7.7) [17] supplemented with 0.1% casamino acids, 38 mM glycerol, and the indicated concentrations of MgCl_2_. *Escherichia coli* DH5α was used as the host for plasmid DNA preparation. Antibiotics were used at the following concentrations: ampicillin, 50 mg/ml; gentamicin, 10 μg/ml; and kanamycin, 50 μg/ml.

### Plasmid Construction

For insertion of the T7 RNA polymerase gene into the *Salmonella* chromosome, the plasmid pGRG36-T7 RNAP was constructed as follows. DNA fragments corresponding to the T7 RNA polymerase gene were amplified by PCR using the primer pair SPU284/SPU285 and plasmid pJLG038 [11] as the template. After purification, the PCR products were digested with XmaI and XhoI and cloned into the pGRG36 plasmid digested with the same enzymes. As the pGRG36 transposon system exhibits leaky expression, all cloning was performed in the presence of 0.2% glucose.

### Measurement of Bacterial Growth

Growth of *Salmonella* containing chromosomally integrated T7 RNAP was measured. As a control, the wild-type *Salmonella* 14028s strain was used. For growth tests, strains were streaked onto solid LB plates and incubated at 37°C. A single colony was then inoculated into 2 ml of LB or N-minimal medium containing 10 mM Mg^2+^ and incubated at 37°C. A 1/100 dilution of pre-cultured samples was inoculated into 15 ml LB or N-minimal medium and cultured with shaking at 37°C. For growth in N-minimal medium containing 10 μM Mg^2+^, pre-cultured samples grown in N-minimal medium were washed twice with N-minimal medium without Mg^2+^ before inoculation. Then, the OD_600_ value was measured every hour.

### Protein analysis by SDS-PAGE and immunoblotting

Overnight cultures were diluted 1:100 into 10 ml of LB medium with or without 1 mM IPTG and grown for 3 h. Cells were normalized by measuring OD_600_. Crude extracts were prepared in TBS (Tris-buffered saline) buffer by sonication, electrophoresed on 12% SDS-polyacrylamide gels. For Coomassie staining, the gel was stained with EZ-gel staining solution (DoGenBio: DG-GS1000, Korea) for 1 h at room temperature.

For Western blot analysis, proteins were transferred to nitrocellulose membranes after electrophoresis. Fur and GFP were detected using anti-Fur polyclonal and anti-GFP monoclonal antibodies (Roche: 1814460001, Switzerland), respectively. The blots were incubated with above antibodies overnight at 4°C, followed by incubation with horseradish peroxidase–conjugated anti-rabbit (Thermo Fisher Scientific: 31460, USA) or anti-mouse IgG (Sigma-Aldrich: NA931V, USA) secondary antibodies (1:10,000 dilution) for 1 h. Signals were detected using SuperSignal^®^ West Femto Maximum Sensitivity Substrate (ThermoFisher: 34095). The data are representative of at least two independent experiments, which gave similar results.

### Quantification of GFP Fluorescence Using a Plate Reader

Overnight bacterial cultures were diluted 1:100 into 10 ml of N-minimal medium containing 0.01 mM Mg²+ and grown for 3 h. 1 mM of IPTG was then added, and cultures were incubated for an additional hour. For measuring GFP expression, cells were aliquoted into a 96-well black/clear-bottom plate (ThermoFisher), GFP fluorescence and OD_600_ were determined using a Synergy H1 plate reader (BioTek Instruments, USA) by measuring fluorescence at 485 nm excitation and 535 nm emission (GFP) and absorbance at 600 nm (OD_600_). GFP expression levels were calculated by dividing the GFP fluorescence values by the OD_600_.

### Flow Cytometry

Macrophage-like J774A.1 cells were cultured in Dulbecco’s Modified Eagle Medium (DMEM) supplemented with 10% (v/v) fetal bovine serum (FBS) at 37°C in a 5% CO_2_ atmosphere. Prior to infection, 5 × 10^5^ cells were seeded into 24-well plates and incubated for 20 h. *Salmonella* strains harboring either pFCcGi (a dual-fluorescence reporter plasmid expressing constitutive mCherry and inducible GFP) were grown overnight in LB medium. Bacterial cultures were used to infect macrophages at a multiplicity of infection (MOI) of 10. Plates were centrifuged at 1,500 rpm for 5 min at room temperature and incubated for 30 min to facilitate bacterial uptake. Extracellular bacteria were removed by washing three times with phosphate-buffered saline (PBS) and subsequently killed by incubating cells in DMEM supplemented with 10% FBS and 120 μg/ml gentamicin for 1 h. The medium was then rep*_lac_*ed with fresh DMEM containing 12 μg/ml gentamicin, and infection was continued at 37°C. At indicated time points, macrophages were washed and lysed with PBS containing 0.1% Triton X-100. Intracellular bacterial fluorescence was assessed using a NovoCyte flow cytometer (ACEA Biosciences, USA). mCherry and GFP signals were excited at 488 nm, with emissions collected at 615 nm (red) and 530 nm (green), respectively. Data acquisition and analysis were performed using NovoExpress software (ACEA Biosciences) [18].

### Macrophage Survival Assay

Intracellular survival assays were performed using the J774A.1 murine macrophage-like cell line as previously mentioned [19]. A total of 5 × 105 macrophages were seeded in 24-well plates containing Dulbecco’s Modified Eagle’s Medium (DMEM) supplemented with 10% fetal bovine serum (FBS) and incubated at 37°C with 5% CO_2_. Overnight-grown bacteria were added to the macrophages at a multiplicity of infection (MOI) of 10. Plates were centrifuged at 1,500 rpm for 5 min at room temperature and incubated for an additional 30 min to facilitate bacterial uptake. Extracellular bacteria were removed by washing wells twice with phosphate-buffered saline (PBS), followed by incubation in DMEM supplemented with 10% FBS and 120 μg/ml gentamicin for 1 h to kill remaining extracellular bacteria. To determine intracellular bacterial counts at 1 h post-infection, macrophages were lysed with PBS containing 0.1% Triton X-100, and serial dilutions of the lysates were plated on Luria–Bertani (LB) agar. For 22 h time points, after the initial 1 h gentamicin treatment, the medium was rep*_lac_*ed with fresh DMEM containing 12 μg/ml gentamicin, and incubation continued at 37°C. At 22 h, cells were lysed as described above, and viable intracellular bacteria were quantified by plating. Percentage survival was calculated by dividing colony-forming units (CFUs) recovered at 22 h by those recovered at 1 h. All experiments were performed in duplicate and repeated independently at least three times.

### Mouse Virulence Assay

Six- to eight-week-old female C3H/HeN mice were inoculated intraperitoneally with approximately 10^3^ colony-forming units (CFU) of *Salmonella* strains. Mouse survival was monitored for 21 days. Virulence assays were repeated two times with consistent outcomes, and the presented data represent groups of ten mice per strain. All animals were housed in a temperature- and humidity-controlled facility with a 12 h light/12 h dark cycle. Experimental procedures were approved by the Korea University Institutional Animal Care and Use Committee (KUIACUC-2024-0054).

## Results

### Integration of T7 RNA Polymerase into the *Salmonella* Chromosome by Tn7 Transposon

**Fig. 1:**
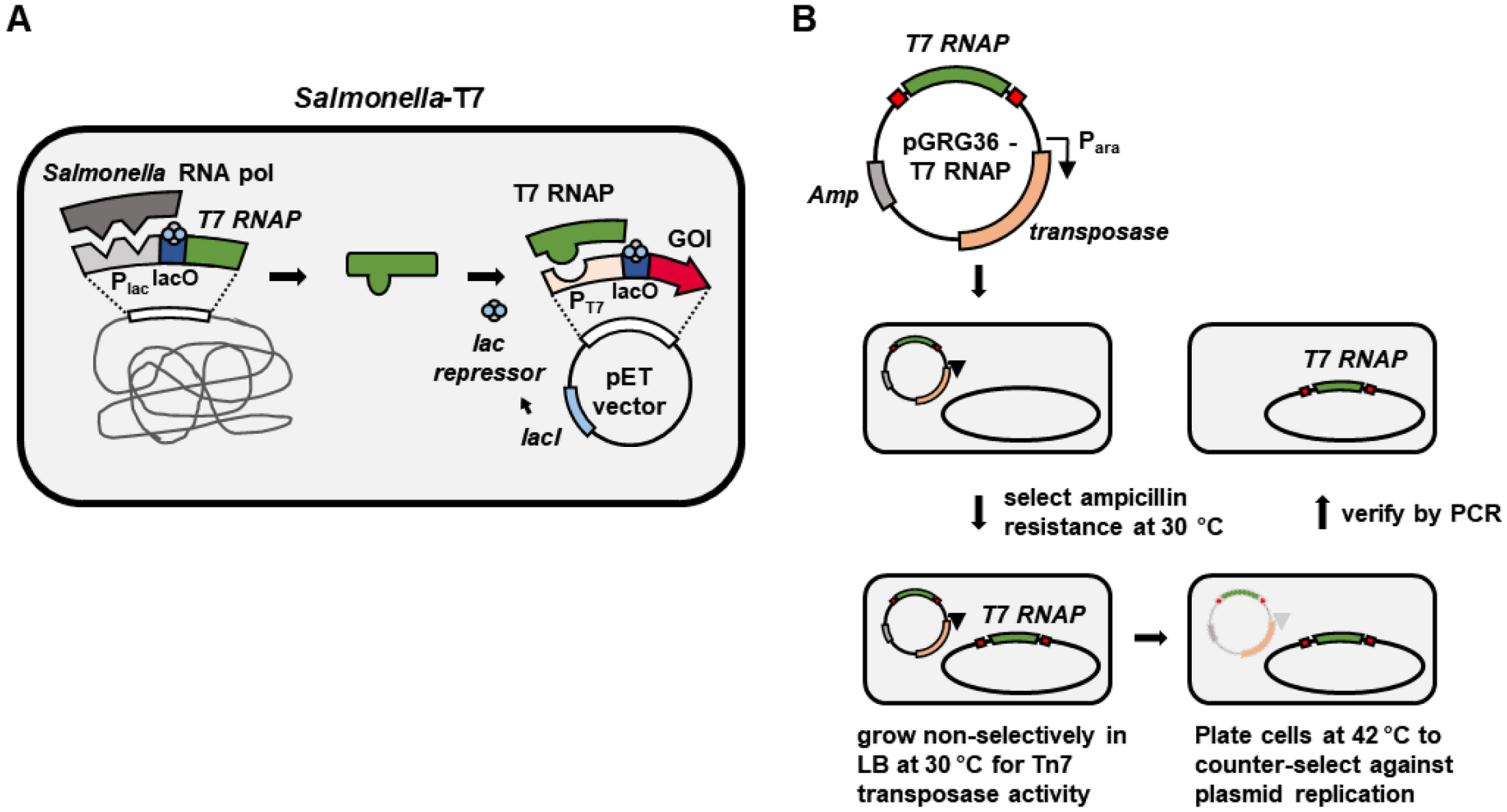
Schematic overview of the engineered *Salmonella*-T7 strain and its construction. (**A**) T7 RNA polymerase (T7 RNAP) was integrated into the *Salmonella* chromosome under the control of the lac promoter (Plac) and lac operator (lacO), allowing IPTG-inducible expression. Upon induction, T7 RNA polymerase is expressed from the lac promoter by *Salmonella* RNA polymerase. Then, T7 RNAP drives transcription from T7 promoters (PT7) present on pET vectors, enabling the expression of genes of interest (GOI) in a pathogenic *Salmonella* background. The system maintains tight regulation through the LacI repressor and supports compatibility with existing T7-based expression constructs. (**B**) Construction strategy of the *Salmonella*-T7 strain. The pGRG36-T7 RNAP plasmid carrying T7 RNA polymerase and a temperature-sensitive origin was transformed into wild-type *Salmonella* and selected for ampicillin resistance at 30°C. Cells were then grown non-selectively at 30°C to induce Tn7 transposition activity, followed by counter-selection at 42°C to eliminate plasmid replication. Successful chromosomal integration of T7 RNA polymerase was verified by sequencing.

To enable the use of T7 promoter-driven expression systems in *S. enterica* subsp. *enterica* serovar Typhimurium strain 14028s, we engineered a strain with chromosomally integrated T7 RNA polymerase under the control of the lac promoter and operator (p*_lac_*/*lacO*), allowing IPTG-inducible expression (Fig. 1A). This strategy replicates the core regulatory logic of the *E. coli* BL21(DE3) system but in a pathogenic *Salmonella* background. The integration was performed using the pGRG36-T7 RNAP plasmid, which carries the T7 RNA polymerase gene and a temperature-sensitive replication origin (Fig. 1B) [20]. Transformation of wild-type *Salmonella* Typhimurium 14028s with this plasmid and selection at 30°C for ampicillin resistance induced expression of the Tn7 transposases, resulting in recombination of the T7 RNA polymerase gene into the *Salmonella* chromosome. Non-selective growth at 30°C facilitated site-specific insertion of the T7 RNA polymerase gene at the *att*Tn7 site downstream of *glmS*. Counter-selection at 42°C eliminated the temperature-sensitive plasmid, and chromosomal integration was confirmed by PCR and sequencing. This genetic engineering approach generated a strain (*Salmonella*-T7) capable of expressing T7 RNA polymerase without the need for plasmid maintenance or antibiotic selection, providing a stable system for T7 promoter-driven expression in *Salmonella*.

### T7 RNA Polymerase in *Salmonella* Tightly Regulates IPTG-Inducible GFP Expression

**Fig. 2:**
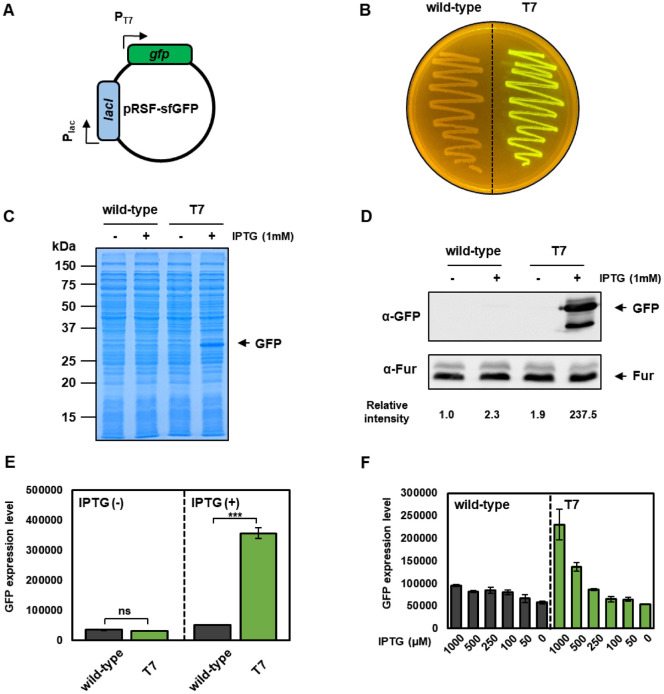
*Salmonella*-T7 tightly regulates IPTG-inducible GFP expression. (**A**) Schematic representation of the reporter plasmid pRSF-sfGFP, which expresses GFP under the control of a T7 promoter (*P*_T7_) and is regulated by the LacI repressor driven by the *P*_lac_ promoter. (**B**) Plate-based visualization of GFP expression. Wild-type *Salmonella* and T7-integrated strains harboring pRSF-sfGFP were streaked on LB agar plates containing 1 mM IPTG. (C, D) Coomassie-stained SDS-PAGE gel (**C**) and Western blot (**D**) showing GFP expression from the pRSF-sfGFP vector. Cells were grown for 3 h in LB medium in the presence and absence of 1mM IPTG. GFP proteins were detected using anti-GFP antibodies, and Fur proteins (loading control) were detected using anti-Fur antibodies. Relative intensity values are shown below each lane. Band intensities were quantified using ImageJ. For each band, background-subtracted intensities were measured. GFP band intensities were normalized to the corresponding Fur loading control for each lane to obtain normalized intensity values (GFP/Fur). All values were further normalized to the wild-type sample without IPTG induction (set to 1) to obtain relative intensity values. (**E**, **F**) GFP fluorescence measurements demonstrating IPTG-inducible expression from pRSF-sfGFP. Cells were grown for 3 h in N-minimal medium containing 10 mM Mg^2+^, and 1 mM IPTG was added for an additional hour. GFP expression levels were calculated by dividing GFP fluorescence by OD_600_. (**E**) Quantification of GFP fluorescence in the presence or absence of IPTG. Error bars represent standard deviation (*n* = 3). Statistical analysis was performed using an unpaired *t*-test (***p < 0.001; ns, not significant). (**F**) Dose-dependent GFP expression in wild-type and T7 strains in response to increasing IPTG concentrations. The T7 strain showed robust, titratable induction of GFP, while the wild-type background showed no significant IPTG-dependent change. Data represent means ± SD from three biological replicates.

We next assessed whether the integrated T7 RNA polymerase is functional and capable of driving expression from T7 promoters. The pRSF-sfGFP reporter plasmid, a pET-based vector containing GFP under T7 promoter control [21], was introduced into both *Salmonella*-T7 and wild-type strains (Fig. 2A). When streaked onto LB agar plates containing 1 mM IPTG, only *Salmonella*-T7 colonies exhibited bright green fluorescence visible under UV illumination, while wild-type colonies remained non-fluorescent (Fig. 2B).

To further quantify expression, cultures were grown in LB broth in the presence or absence of 1 mM IPTG, and total protein extracts were analyzed by SDS-PAGE and Western blotting. In Coomassie-stained gels, a strong band corresponding to GFP (~27 kDa) was detected in *Salmonella*-T7 samples with IPTG addition but not in uninduced or wild-type samples (Fig. 2C). Western blotting using anti-GFP antibodies confirmed robust GFP production in *Salmonella*-T7 following IPTG induction (Fig. 2D). These results indicate that expression is tightly regulated, with minimal background in the absence of IPTG.

Fluorescence measurements in N-minimal medium supplemented with 10 mM Mg^2+^ supported these findings. GFP expression in *Salmonella*-T7 increased approximately 10-fold upon IPTG induction, while wild-type strains showed no significant change (Fig. 2E). Dose-dependent GFP expression demonstrated that *Salmonella*-T7 supports tunable GFP expression across IPTG concentrations from 50 μM to 1 mM (Fig. 2F). Collectively, these results establish that chromosomally expressed T7 RNA polymerase in *Salmonella*-T7 enables strong and tightly controlled expression from T7 promoters.

### Insertion of T7 RNA Polymerase Does Not Affect Bacterial Growth

**Fig. 3:**
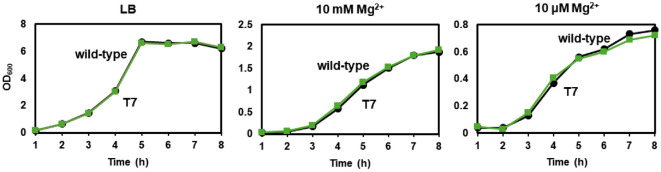
Salmonella with T7 polymerase does not exhibit a growth defect. Wild-type *Salmonella enterica* serovar Typhimurium 14028s and a strain harboring a chromosomal integration of T7 RNA polymerase (T7) were grown in LB medium (left), or N-minimal medium supplemented with either 10 mM Mg^2+^ (center) or 10 μM Mg^2+^ (right). Cultures were incubated at 37°C with shaking, and optical density at 600 nm (OD_600_) was measured hourly.

To determine whether chromosomal integration of T7 RNA polymerase impacts bacterial fitness, we compared the growth of *Salmonella*-T7 and wild-type 14028s in various culture conditions. Growth curves in LB medium, a rich medium for *Salmonella*, demonstrated comparable growth between the two strains, with OD_600_ values reaching stationary phase at approximately the same time (Fig. 3, left panel). To assess growth under nutrient-limiting conditions, strains were cultured in N-minimal medium supplemented with either 10 mM or 10 μM Mg^2+^. Again, no significant differences in growth rates or final cell densities were observed between *Salmonella*-T7 and the wild-type (Fig. 3, center and right panels). Together, these results indicate that chromosomally expressed T7 RNA polymerase does not impose a detectable metabolic burden or growth defect in either rich or minimal media.

### *Salmonella*-T7 Strain Retains Virulence in Macrophages and Mice

**Fig. 4:**
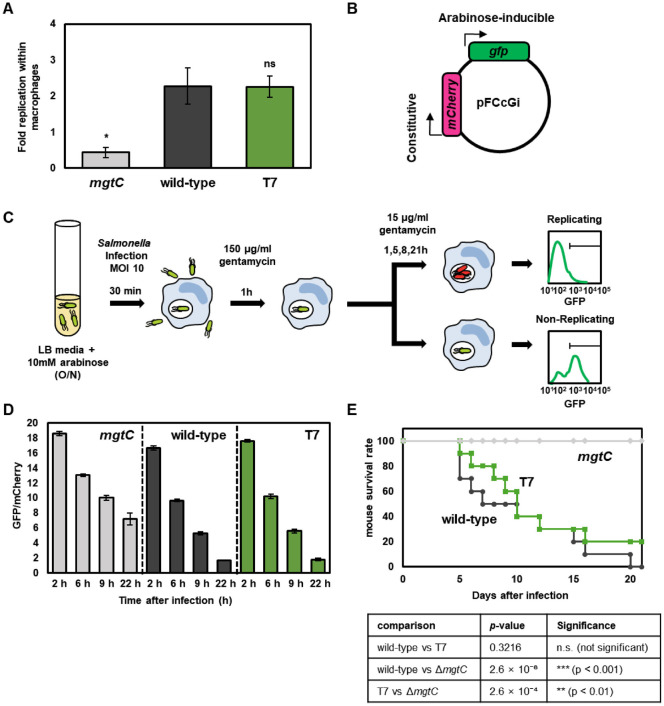
T7 RNA polymerase integration does not affect intracellular survival or virulence of *Salmonella*. (**A**) Intracellular survival of wild-type *Salmonella* (14028s) and engineered *Salmonella*-T7 (SW396) strains at 22 h post-infection (T22) in J774A.1 macrophages. Intracellular bacterial counts were determined at 1 h (T1) and 22 h (T22) post-infection, and fold replication was calculated as CFU at T22 divided by CFU at T1. Data represent means ± SD from three independent infections. An asterisk indicates a statistically significant difference (*p* = 0.018) between Δ*mgtC* and wild-type; ns indicates no significant difference between wild-type and *Salmonella*-T7 (unpaired *t*-test). (**B**) Schematic representation of the pFCcGi vector. (**C**) Schematic representation of the flow cytometry-based assay to measure non-replicating *Salmonella* inside macrophages using bacteria carrying pFCcGi. (**D**) Quantification of GFP/mCherry ratios for Δ*mgtC*, wild-type, and *Salmonella*-T7 strains at 2, 6, 9, and 22 h post-infection. Bars represent mean values ± SD from three independent experiments. (**E**) Survival of C3H/HeN mice (*n* = 10 per group) infected intraperitoneally with 10^3^ CFU of either wild-type *Salmonella* or *Salmonella*-T7. Mouse survival was monitored for 21 days post-infection. Statistical significance was evaluated by log-rank (Mantel–Cox) test. The table summarizes pairwise comparisons: wild-type vs T7, *p* = 0.3216 (n.s.); wild-type vs Δ*mgtC*, *p* = 2.6 × 10^-6^ (***, *p* < 0.001); T7 vs Δ*mgtC*, *p* = 2.6 × 10^-4^ (**, *p* < 0.01).

To investigate whether chromosomal integration of T7 RNA polymerase affects *Salmonella* virulence, we first performed macrophage survival assays. J774A.1 macrophages were infected with *Salmonella*-T7, wild-type 14028s, or the Δ*mgtC* mutant, which has lost pathogenic activity [16], and intracellular bacterial replication was quantified. At 22 h post-infection, fold replication (CFU T22/T1) of *Salmonella*-T7 was comparable to the wild-type, whereas Δ*mgtC* mutants showed significantly reduced survival (Fig. 4A).

To determine whether *Salmonella*-T7 impacts intracellular bacterial dynamics, we utilized the pFCcGi plasmid, a reporter system that measures non-replicating bacteria during host infection via a fluorescence dilution assay. This dual-fluorescence reporter constitutively expresses mCherry and arabinose-inducible GFP (Fig. 4B). Following macrophage infection, bacteria were analyzed by flow cytometry to determine replication status based on GFP dilution relative to the constitutive mCherry signal (Fig. 4C). Non-replicating bacteria maintain high GFP/mCherry ratios, while replicating bacteria exhibit decreased GFP signals due to dilution. At 2, 6, 9, and 22 h post-infection, Δ*mgtC* mutants consistently maintained high GFP/mCherry ratios, indicating non-replication. However, *Salmonella*-T7 strains displayed progressive GFP dilution over time, indicating active replication similar to the wild-type (Figs. 4D and S1). These findings highlight that introduction of T7 RNA polymerase into *Salmonella* does not alter bacterial physiology during host infection.

*In vivo* virulence was assessed by intraperitoneal infection of C3H/HeN mice with 10^3^ CFU of *Salmonella*-T7, wild-type, or Δ*mgtC*. Mouse survival was monitored over 21 days post-infection. *Salmonella*-T7 and wild-type strains exhibited similar lethality profiles, with no significant differences in survival rates (Fig. 4E). Together, these data demonstrate that integration of T7 RNA polymerase does not attenuate *Salmonella* virulence *in vitro* or *in vivo*.

## Discussion

In this study, we developed and characterized a novel *Salmonella enterica* Typhimurium strain (*Salmonella*-T7) engineered to express T7 RNA polymerase from the chromosome under the control of the lac promoter by using a transposon insertion system [20]. This strain enables IPTG-inducible expression of heterologous proteins from T7 promoter-based vectors, such as the widely used pET series, directly in a pathogenic *Salmonella* background. Using the pRSF-sfGFP reporter plasmid, we observed strong GFP induction upon IPTG treatment with minimal background expression in its absence, both in rich and minimal media (Fig. 2). The ability to achieve dose-dependent, tunable expression further enhances the utility of *Salmonella*-T7 for applications requiring precise control over gene expression.

Earlier attempts to integrate T7 RNA polymerase in *Salmonella*, such as the *nirB*-regulated system in *S. Typhi* [22] and the *araC*-P_BAD_ system in *S*. Typhimurium [23], provided proof-of-concept for expressing proteins in *Salmonella*. The broad-host-range approach by Hoang *et al*. (2007) using mariner transposons [24] also demonstrated the potential for integrating T7 RNAP into diverse bacteria, including *Salmonella*. Although previous systems using *nirB* [23] or P_BAD_ [24] promoters demonstrated reporter gene activity through methods such as northern blotting or β-galactosidase assays, they did not confirm heterologous protein expression at the protein level (*e.g.*, by SDS-PAGE or Western blot analysis) or assess virulence in host infection models. In contrast, our system utilizes site-specific Tn7 integration at *att*Tn7, uses the well-characterized *p*_lac_/*lacO* promoter for tight IPTG-inducible regulation, and supports seamless expression from widely used pET vectors. Importantly, we show for the first time that chromosomal T7 RNA polymerase expression does not attenuate bacterial virulence *in vitro* or *in vivo* (Figs. 4 and S1), positioning *Salmonella*-T7 as a robust platform for pathogenicity studies and synthetic biology applications. While our *in vivo* analysis focused on survival outcomes and did not include organ bacterial burden measurements, the consistent lethality profiles support the conclusion that virulence was not notably affected. Future studies incorporating organ CFU quantification may provide additional resolution into tissue-specific infection dynamics.

The development of *Salmonella*-T7 overcomes several limitations of previous expression systems. Single-vector systems in native hosts often suffer from leaky expression, whereas bipartite systems employing two plasmids require dual antibiotic selection, which could be undesirable for studies of pathogenic bacteria. Our chromosomal integration strategy eliminates the need for plasmid maintenance and additional selection markers, enabling stable and reproducible gene expression studies in pathogenic backgrounds. Furthermore, this system can be applied to study virulence factors and host-pathogen interactions under native physiological conditions. By enabling direct expression of heterologous or engineered proteins in a pathogenic context, *Salmonella*-T7 provides a unique tool for examining bacterial physiology, secretion systems, and immune evasion strategies. Additionally, it could facilitate the production of antigens or vaccines where maintaining the pathogen’s native virulence and regulatory networks is crucial. As *Salmonella* serves as a platform for live attenuated vaccine development due to its ability to invade host cells and target cancer cells [25-28], our findings highlight that this engineered strain, with its precise regulatory control, could contribute to the development of *Salmonella* as an effective vaccine carrier. Moreover, this approach could be adapted to other non-model Gram-negative pathogens, expanding its utility in microbial pathogenesis research.

In conclusion, we present *Salmonella*-T7 as a powerful and versatile platform for T7 promoter-driven expression in *S. enterica*. By enabling robust, IPTG-inducible expression of heterologous proteins directly from widely used pET vectors, this system overcomes key limitations of previous approaches and facilitates the seamless transfer of constructs optimized in *E. coli*. Its ability to maintain full virulence while supporting tightly regulated gene expression makes it highly suitable for both *in vitro* and *in vivo* studies of *Salmonella* biology. Furthermore, *Salmonella*-T7 provides a valuable tool for dissecting bacterial physiology and host-pathogen interactions under native conditions, while also offering potential applications in vaccine development and synthetic biology.

## Supplemental Materials

Supplementary data for this paper are available on-line only at http://jmb.or.kr.



## Figures and Tables

**Table 1:** Bacterial strains, plasmids, and oligonucleotides used in this study.