# Supplementary material for: A Chromosomally Integrated T7 RNA Polymerase Enables T7-Derived Expression in Salmonella enterica without Compromising Virulence
Source: J Microbiol Biotechnol. 2025 Nov 18;35:e2509023. doi: 10.4014/jmb.2509.09023 (PMC12640770; doi:10.4014/jmb.2509.09023)
Supplement: Supplementary file 1 [file jmb-35-e2509023-supple.pdf]

J. Microbiol. Biotechnol. 2025. 35: e259023

https://doi.org/10.4014/jmb.2509.09023

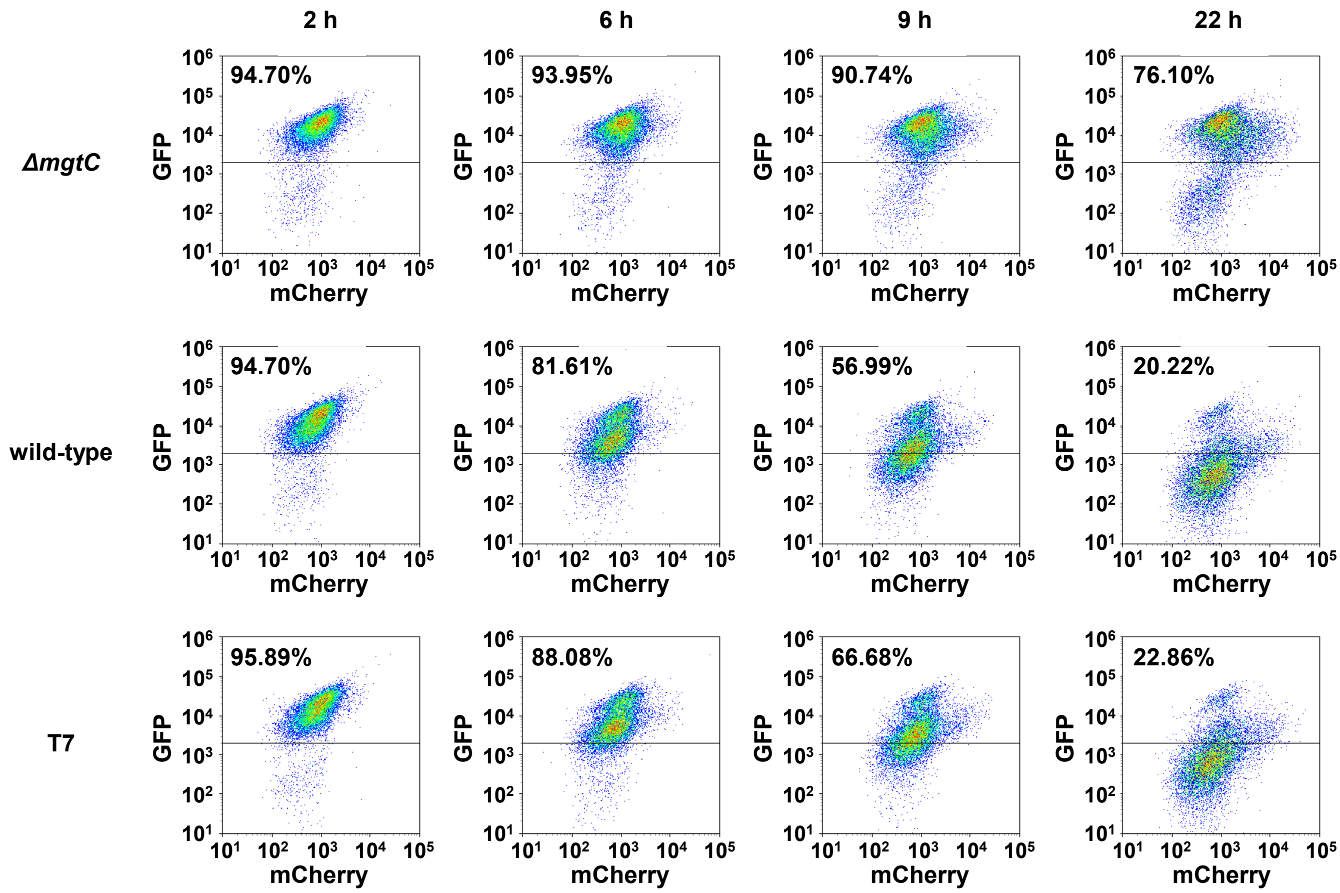

Fig. S1. T7 RNA polymerase insertion does not affect the formation of non-replicating *Salmonella* inside macrophages.

Quantification of GFP/mCherry ratios for *ΔmgtC*, wild-type, and *Salmonella*-T7 strains at 2, 6, 9, and 22 h post-infection. Cells were gated using FSC/SSC dot plots to select live bacteria, followed by FSC-H versus FSC-A gating to exclude doublets and select single cells. mCherry/GFP plots were then analyzed to identify bacteria emitting high levels of green fluorescence (GFP > 2000).
